# A Preclinical Study on Brugada Syndrome with a CACNB2 Variant Using Human Cardiomyocytes from Induced Pluripotent Stem Cells

**DOI:** 10.3390/ijms23158313

**Published:** 2022-07-27

**Authors:** Rujia Zhong, Theresa Schimanski, Feng Zhang, Huan Lan, Alyssa Hohn, Qiang Xu, Mengying Huang, Zhenxing Liao, Lin Qiao, Zhen Yang, Yingrui Li, Zhihan Zhao, Xin Li, Lena Rose, Sebastian Albers, Lasse Maywald, Jonas Müller, Hendrik Dinkel, Ardan Saguner, Johannes W. G. Janssen, Narasimha Swamy, Yannick Xi, Siegfried Lang, Mandy Kleinsorge, Firat Duru, Xiaobo Zhou, Sebastian Diecke, Lukas Cyganek, Ibrahim Akin, Ibrahim El-Battrawy

**Affiliations:** 1 First Department of Medicine, Faculty of Medicine, University Medical Centre Mannheim (UMM), University of Heidelberg, 68167 Mannheim, Germany; rujia.zhong@medma.uni-heidelberg.de (R.Z.); schimanski.t@gmail.com (T.S.); feng.zhang@medma.uni-heidelberg.de (F.Z.); or lh6402196@126.com (H.L.); alyssa.hohn@web.de (A.H.); qiang.xu@medma.uni-heidelberg.de (Q.X.); mengying.huang@medma.uni-heidelberg.de (M.H.); zhenxing.liao@medma.uni-heidelberg.de (Z.L.); lin.qiao@medma.uni-heidelberg.de (L.Q.); zhen.yang@medma.uni-heidelberg.de (Z.Y.); yingrui.li@medma.uni-heidelberg.de (Y.L.); zhihan.zhao@medma.uni-heidelberg.de (Z.Z.); xin.li@medma.uni-heidelberg.de (X.L.); roselena1996@gmail.com (L.R.); sebastian9876@googlemail.com (S.A.); lasse-maywald@web.de (L.M.); jonasnelsonmueller@googlemail.com (J.M.); hendrik.dinkel@yahoo.de (H.D.); yannick.xi@medma.uni-heidelberg.de (Y.X.); siegfried.lang@umm.de (S.L.); ibrahim.akin@umm.de (I.A.); 2 DZHK (German Center for Cardiovascular Research), Partner Site, 68167 Mannheim, Germany; narasimha.swamy@mdc-berlin.de (N.S.); mandy.kleinsorge@gwdg.de (M.K.); sebastian.dieck@mdc-berlin.de (S.D.); lukas.cyganek@gwdg.de (L.C.); 3 Key Laboratory of Medical Electrophysiology of Ministry of Education and Medical Electrophysiological Key Laboratory of Sichuan Province, Institute of Cardiovascular Research, Southwest Medical University, Luzhou 646000, China; 4 Department of Cardiology, University Heart Center Zurich, Rämistrasse 100, 8091 Zürich, Switzerland; ardan.saguner@usz.ch (A.S.); first.duru@usz.ch (F.D.); 5 Department of Human Genetics, Institute of Human Genetics, University of Heidelberg, 69120 Heidelberg, Germany; johannes.jannsen@uni-heidelberg.de; 6 Max Delbrück Center for Molecular Medicine, 13125 Berlin, Germany; 7 Stem Cell Unit, Clinic for Cardiology and Pneumology, University Medical Center Göttingen, 37075 Göttingen, Germany; 8 Bergmannsheil University Hospitals, Ruhr University of Bochum, 44789 Bochum, Germany; ibrahim.elbattrawy2006@gmail.com

**Keywords:** Brugada syndrome, arrhythmias, human-induced pluripotent stem cell-derived cardiomyocytes, CACNB gene

## Abstract

**Aims:** Some gene variants in the sodium channels, as well as calcium channels, have been associated with Brugada syndrome (BrS). However, the investigation of the human cellular phenotype and the use of drugs for BrS in presence of variant in the calcium channel subunit is still lacking. **Objectives:** The objective of this study was to establish a cellular model of BrS in the presence of a CACNB2 variant of uncertain significance (c.425C > T/p.S142F) using human-induced pluripotent stem cell-derived cardiomyocytes (hiPSC-CMs) and test drug effects using this model. **Methods and results:** This study recruited cells from a patient with Brugada syndrome (BrS) and recurrent ventricular fibrillation carrying a missense variant in CACNB2 as well as from three healthy independent persons. These cells (hiPSC-CMs) generated from skin biopsies of healthy persons and the BrS patient (BrS-hiPSC-CMs) as well as CRISPR/Cas9 corrected cells (isogenic control, site-variant corrected) were used for this study. The hiPSC-CMs from the BrS patient showed a significantly reduced L-type calcium channel current (I_Ca-L_) compared with the healthy control hiPSC-CMs. The inactivation curve was shifted to a more positive potential and the recovery from inactivation was accelerated. The protein expression of CACNB2 of the hiPSC-CMs from the BrS-patient was significantly decreased compared with healthy hiPSC-CMs. Moreover, the correction of the CACNB2 site-variant rescued the changes seen in the hiPSC-CMs of the BrS patient to the normal state. These data indicate that the CACNB2 gene variant led to loss-of-function of L-type calcium channels in hiPSC-CMs from the BrS patient. Strikingly, arrhythmia events were more frequently detected in BrS-hiPSC-CMs. Bisoprolol (beta-blockers) at low concentration and quinidine decreased arrhythmic events. **Conclusions:** The CACNB2 variant (c.425C > T/p.S142F) causes a loss-of-function of L-type calcium channels and is pathogenic for this type of BrS. Bisoprolol and quinidine may be effective for treating BrS with this variant.

## 1. Introduction

The Brugada syndrome (BrS) is an inherited channelopathy with the risk of life-threatening arrhythmias and sudden cardiac death (SCD). Patients with BrS can suffer from several symptoms, e.g., syncope, seizures, and nocturnal agonal breathing due to polymorphic ventricular tachycardia (PVT) or ventricular fibrillation (VF). In case of persistence of arrhythmias, SCD can be common. To date, syncope or SCD ranges from 17% to 42% [1]. However, recently published data reported a significantly lower proportion of SCD as the first symptom (4.6%) and a lower incidence of recurrent arrhythmia during follow-up (5%) [2]. Symptoms of BrS often occur during adulthood, at mean age of 41 years but patients may also suffer from symptoms in childhood. Of note, syncope in patients could be benign, related to neurally-mediated syncope, and distinguishing this syncope from the real, malignant syncope can be complicated [3,4]. Therefore, a critical diagnostic and treatment approach is required in these cases.

The ECG of the patient shows a concave (coved type, type 1) ST-segment elevation ≥ 2 mm in ≥1 right precordial lead, followed by a negative T-wave, or a convex (saddle-back type, type 2) ST-segment elevation ≥ 0.5 mm (generally ≥ 2 mm) in ≥1 right precordial lead followed by a positive T-wave [5]. The worldwide prevalence of this disease is about 0.05% [6]. The incidence rate for males is higher than that for females [5,7]. The arrhythmia of BrS happens usually at rest or during sleep and is a leading cause of death, excluding accidents, in men < 40 years old [8]. The unmasking of BrS can be caused by fever or sodium channel blockers, therefore, statement papers recommended patients to avoid sodium channel blockers and infections [2].

Since the BrS was first described in 1992, numerous studies have been conducted to uncover the molecular basis and underlying pathomechanisms inducing arrhythmia and SCD. In early 1998, the first gene variant associated with BrS was found in the cardiac sodium channel SCN5A [9]. So far, more than 500 rare variants located in 43 genes have been reported to be BrS-associated [10]. Most of these genes encode the sodium, potassium, and calcium channels or proteins that may modulate these ion channels. The most frequently detected variants, in about 21% of BrS patients, are variants in the cardiac sodium channel gene SCN5A. In addition, other gene variants, including variants in the subunits of the L-type calcium channel, have been reported [11].

The cardiac L-type calcium channel is encoded by a protein complex of three subunits, α_1_, β, and α_2_δ. Although the pore-forming Ca_v_1.2 α_1_-subunit (encoded by CACNA1C) is the dominating functional part of I_Ca-L_, the β-(Ca_v_β2b) subunit, encoded by CACNB2 gene, is a regulatory subunit that may affect the gating kinetics or the trafficking of calcium channels. A variant in either the alpha- or beta-subunit may predispose a patient to BrS [12,13].

In spite of rapid advances in understanding the mechanisms and genetic bases of BrS, much less is known about the genotype–phenotype association. Although hundreds of variants in different genes have been detected in BrS patients, experimental proofs for their pathogenic roles remain sparse. Most of the reported functional studies focused on variants in sodium channels, especially in the SCN5A gene. Much less data with respect to functional studies on variants in calcium channels are documented. Clear experimental proofs for the genotype–phenotype correlation of calcium channel gene variants in cardiomyocytes from BrS patients are still lacking.

Taking into account the hurdle for obtaining human ventricular cardiomyocytes and the advantages of human-induced pluripotent stem cell-derived cardiomyocytes (hiPSC-CMs) over other models, hiPSC-CMs could be an attractive alternative for BrS studies, either mechanistic or therapeutic. Different inherited cardiac diseases have been studied using hiPSC-CMs [14,15,16,17,18].

Since (1) hiPSC-CMs can model the cellular phenotype of BrS with SCN5A-variants [18], (2) a model using hiPSC-CMs from BrS patients carrying calcium channel variants is still lacking, (3) no drug-testing was performed in such models and (4) the pathogenic significance of the missense variants in the CACNB2 gene detected in BrS patients is still unclear, we aimed in the present study to establish a cellular model of BrS with a calcium channel variant. We used hiPSC-CMs from a BrS patient with a missense variant of uncertain significance (c.425C > T/p.S142F) in the CACNB2 gene and examined the pathogenic roles of the variant by comparing with hiPSC-CMs of healthy persons. In addition, we used this model for screening drugs as anti-arrhythmic agents (e.g., bisoprolol or quinidine).

## 2. Results

### 2.1. Clinical Data

**Figure 1:**
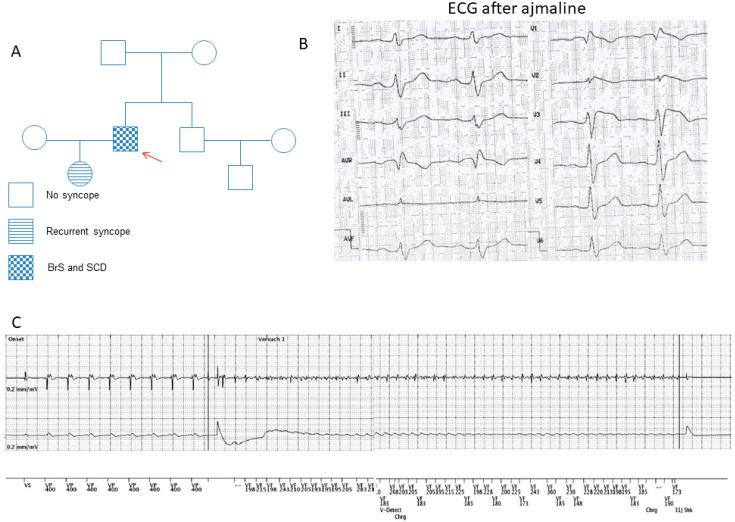
Clinical characteristics of the BrS patient. (**A**) The pedigree of the BrS patient’s family. The patient recruited for this study is marked by the arrow. He has had sudden cardiac death. (**B**) The electrocardiogram (ECG) from the BrS patient shows a classic BrS–ECG pattern after infusion of ajmaline. (**C**) Electrocardiogram of the ICD showing ventricular fibrillation terminated with an appropriate ICD shock after stopping the bisoprolol treatment.

A 55-year-old male patient with BrS carrying a missense variant of uncertain significance (dbSNPrs150528041; NM_000724.4: c.425C > T/p.S142F) in the CACNB2 gene was recruited for this study (Figure 1A). The BrS was diagnosed many years ago after recurrent sudden cardiac arrest (SCA) during resting situations. Using a sodium channel blocker, 40 mg ajmaline, unmasked a type I ECG of BrS (Figure 1B). The patient suffered at least six episodes of SCA. His daughter has suffered from recurrent syncope, but she has rejected clinical checkups. The family pedigree is shown in Figure 1A. A structural cardiac disease was excluded after extensive clinical and instrumental evaluations, including a physical examination, echocardiogram and cardiac MRI. Programmed stimulation in the electrophysiological study induced ventricular fibrillation. The patient received an ICD. Two years after ICD implantation and the termination of the initially started beta-blocker treatment, the patient experienced at least six appropriate ICD shocks (Figure 1C) due to a documented ventricular fibrillation. Therefore, as a possible anti-arrhythmic drug therapy, a beta-blocker treatment (bisoprolol) was restarted. Since then and over a 5-year follow-up period no more ICD shocks were delivered. The next generation sequencing analysis detected no variants in BrS-related genes.

### 2.2. Characterization of Patient Specific hiPSCs and hiPSC-CMs

**Figure 2:**
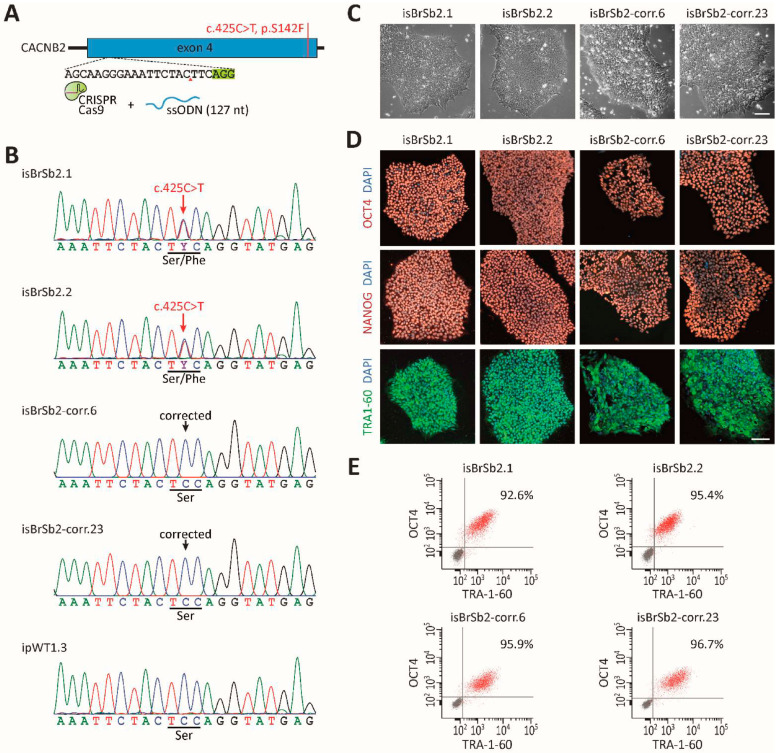
Genetic correction of BrS-hiPSCs by CRISPR/Cas9 genome editing. (**A**) Corrected hiPSCs were generated with a CRISPR guide RNA targeting the CACNB2 exon 4 and a single-stranded oligonucleotide (ssODN) for homology-directed repair. (**B**) Confirmation of genetic correction, assessed by Sanger sequencing of genomic DNA. (**C**) Patients’ and CRISPR-corrected hiPSC lines exhibited a typical human stem cell-like morphology. Scale bar: 100 μm. (**D**) Immunofluorescence staining for key pluripotency markers OCT4, NANOG and TRA1–60 in patients’ and corrected hiPSC lines. Nuclei were counter-stained with DAPI. Scale bar: 100 μm. (**E**) Purity of patient-specific and CRISPR-corrected hiPSC lines was evaluated by the flow cytometry analysis of pluripotency markers OCT4 and TRA1–60. Gray dots represent the negative controls.

We generated hiPSCs from skin fibroblasts of the patient with BrS carrying the variant (c.425C > T/p.S142F) in the CACNB2 gene. Then, we used the CRISPR/Cas9-based genome editing to correct the CACNB2 variant in the generated hiPSCs (Figure 2A,B). Two patient-specific and two CRISPR/Cas9-corrected hiPSC lines were selected and verified for pluripotency and normal karyotype (Figure 2C–E, Figure S1). Three healthy hiPS cell lines, which have been characterized before, were used in the current study. Data from all the healthy cells were combined as healthy controls.

**Figure 3:**
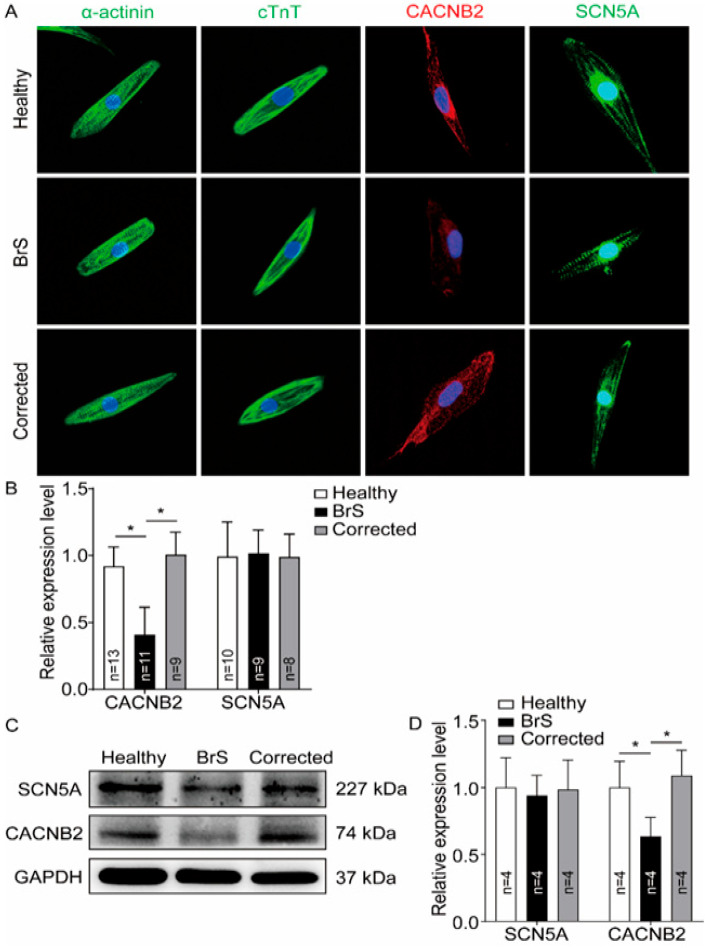
Reduction of CACNB2 protein expression level in hiPSC-CMs from the BrS-patient. Western blot and immunostaining analyses were performed to examine the protein levels of CACNB2 in hiPSC-CMs from healthy donor (Healthy), the patient (BrS) and isogenic control cell line (Corrected). (**A**) Representative immunostaining images. (**B**) Statistical analyses of fluorescence intensity from immunostained cells. (**C**) Representative examples of Western blots of cell lysates from healthy donor cell line (healthy), the patient (BrS) and the CRISPR/Cas9-corrected (Corrected) hiPSC-CMs. (**D**) Statistical analyses of relative expression levels of SCN5A and CACNB2 from Western blot experiments. Data were shown as mean ± standard deviation (SD). Numbers given represent the number of cells (**B**) or experiments (**D**). * *p* < 0.05 versus healthy analyzed by one-way analysis of variance (ANOVA) with Holm–Sidak post-test.

After 8–12 days of differentiation, spontaneously beating cells were observed, and after 50 days of differentiation, cells were applied for the experiments. The successful differentiation of hiPSCs into cardiomyocytes was proven by the expression of different cardio-specific markers (α-actinin and cTnT) analyzed by qPCR and immunostaining (Figure S1, Figure 3A).

### 2.3. L-Type Calcium Channel Current (I_Ca-L_) Was Reduced in BrS-hiPSC-CMs

**Figure 4:**
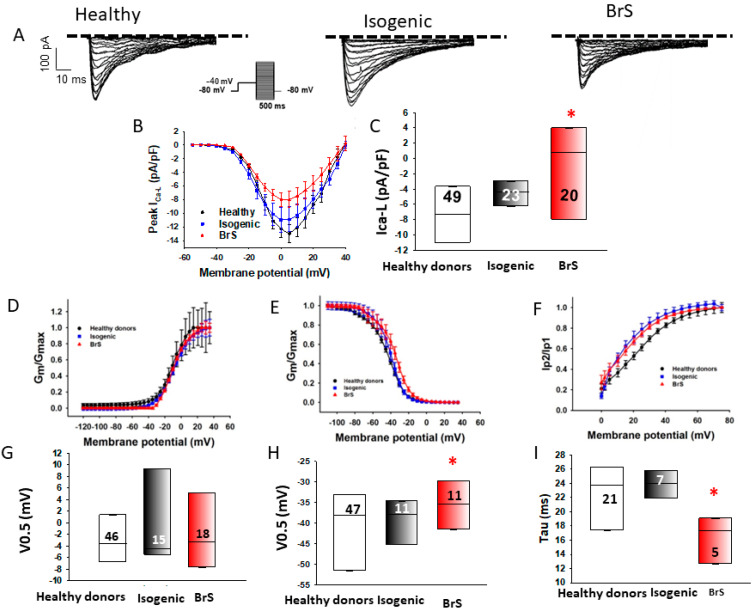
Reduction of L-type calcium channel currents in hiPSC-CMs from the BrS patient. Peak L-type calcium (I_Ca-L_) and sodium channel (I_Na_) currents were analyzed in hiPSC-CMs from healthy donors (Healthy), the BrS patient (BrS) and variant-corrected cells (Isogenic). (**A**) Representative traces of I_Ca-L_ in hiPSC-CMs from healthy donors (Healthy), the BrS patient (BrS) and variant-corrected cells (Isogenic). (**B**) I-V curves of I_Ca-L_ from healthy donors (Healthy), the BrS patient (BrS) and variant-corrected cells (Isogenic). (**C**) Median values of peak I_Ca-L_ at −10 mV from healthy donors (Healthy), the BrS patient (BrS) and variant-corrected cells (Isogenic). (**D**) Activation curves of I_Ca-L_ in hiPSC-CMs from each cell line. (**E**) Inactivation curves of I_Ca-L_ in hiPSC-CMs from each cell line. (**F**) Recovery curves of I_Ca-L_ in hiPSC-CMs from each cell line. (**G**) Median values of the potential at 50% activation (V0.5) of I_Ca-L_ in hiPSC-CMs from each cell line. (**H**) Median values of the potential at 50% inactivation (V0.5) of I_Ca-L_ in hiPSC-CMs from each cell line. (**I**) Median values of the time constants (Tau) of recovery from the inactivation of I_Ca-L_ in hiPSC-CMs from each cell line. Numbers given in (**C**) represent the number of cells for (**B**,**C**). Numbers given in (**F**) represent the number of cells for (**E**,**F**). * *p* < 0.05 versus Healthy according to the analysis of one-way ANOVA with Holm–Sidak post-test.

To examine the possible influence of the variant in CACNB2 on calcium channel functions, the L-type calcium channel current (I_Ca-L_) was measured. The peak I_Ca-L_ was reduced in BrS cardiomyocytes (BrS, −4.6 ± 0.5 pA/pF, versus healthy donors, −8.6 ± 0.9 pA/pF, and isogenic control, −8.7 ± 1.4 pA/pF, Figure 4A–C). Although the activation of I_Ca-L_ was not significantly changed, the inactivation curve in BrS-hiPSC-CMs was shifted to more positive potentials and the recovery from inactivation was accelerated (Figure 4D–I).

### 2.4. Abnormal Action Potentials Were Observed in BrS-hiPSC-CMs

Action potential characterizations are summarized in Figure S2. Action potential amplitude (APA) (BrS 96.7 ± 3.4 mV, healthy donors 137.4 ± 1.7 mV and isogenic control 120.2 ± 1.8 mV) and maximum depolarization velocity (Vmax) (BrS 28.3 ± 1.5 V/s, healthy donors 39.3 ± 1.5 V/s and isogenic control 47.6 ± 6.2 V/s) were significantly decreased in BrS cardiomyocytes (Figure S2A,B). Other action potential parameters, including the resting potential (RP), action potential duration at 50% repolarization (APD50) and action potential duration at 90% repolarization (APD90), were similar in the measured hiPSC-CMs from the BrS patient, the healthy donors and isogenic controls (Figure S2A,D–F).

### 2.5. The Protein Expression of Calcium and Sodium Channels Was Decreased in BrS-hiPSC-CMs

To test whether the reduction of both Ca^2+^ currents in BrS-hiPSC-CMs was linked to changes of protein level of both types of this channel, the gene and protein expression was analyzed by qPCR (mRNA expression), Western blot and immunostaining. The mRNA expression of KCNJ2, KCNH2 and KCNQ1 was reduced and SCN3B mRNA expression was elevated in BrS-hiPSC-CMs (Figure S1A). Strikingly, the protein expression level of CACNB2 but not SCN5A was significantly reduced in BrS-hiPSC-CMs (Figure 3A–D).

### 2.6. Arrhythmia Events Were Increased in BrS-hiPSC-CMs

**Figure 5:**
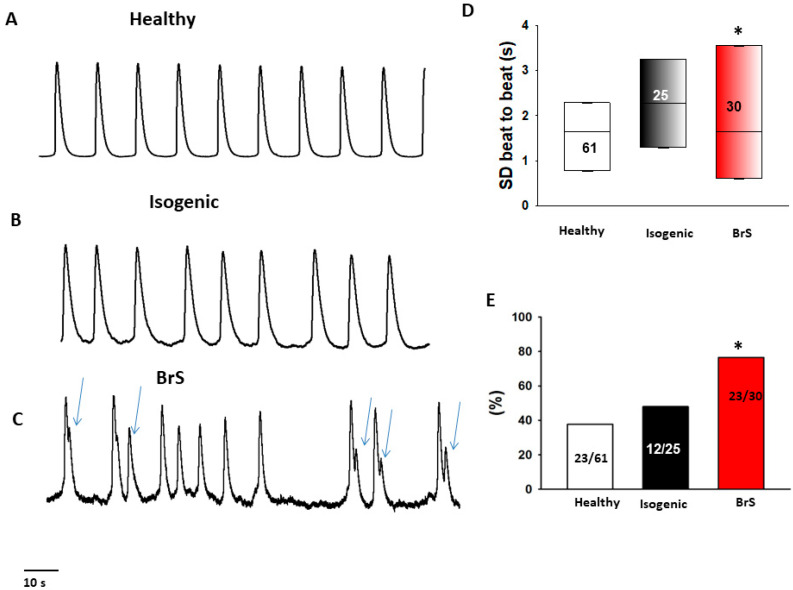
Increased beating variability and arrhythmic events in BrS-hiPSC-CMs. Spontaneous calcium transients were recorded in spontaneously beating hiPSC-CMs from the BrS patient, the healthy donor (healthy) and the CRISPR-corrected cells (isogenic). The beating variability (standard deviation (SD) of cell beating intervals) and the occurrence of arrhythmic events (irregular or triggered beats or EAD-like events) were compared among the three cell groups. (**A**–**C**) Representative traces of calcium transients in cells from each line. Arrhythmic events are marked by arrows. (**D**) Median values of standard deviation (SD) of cell beating intervals. (**E**) Percentage of cells showing arrhythmic events. The numbers given represent number of cells showing arrhythmic events versus total cell number. The numbers given represent number of cells. * *p* < 0.05 versus healthy according to the one-way ANOVA (**D**) or Fisher’s exact test (**E**).

Interestingly, during the measurements of spontaneous calcium transients, BrS cardiomyocytes presented irregular, EAD (early after depolarization)-like arrhythmic events much more frequently than healthy donor cells or isogenic control cells (Figure 5). Among BrS cells, 76% (23/30) of the cells show arrhythmic events, among healthy donor cells the amount was 37% (23/61) and among isogenic control cells it was 48% (12/25) (BrS cells *p* < 0.05, Figure 5E). Moreover, the variation in the beat-to-beat interval time (standard deviations of cell beating interval time) in BrS cells was larger than in healthy and isogenic control cells (Figure 5D).

### 2.7. Drug Testing in BrS-hiPSC-CMs

**Figure 6:**
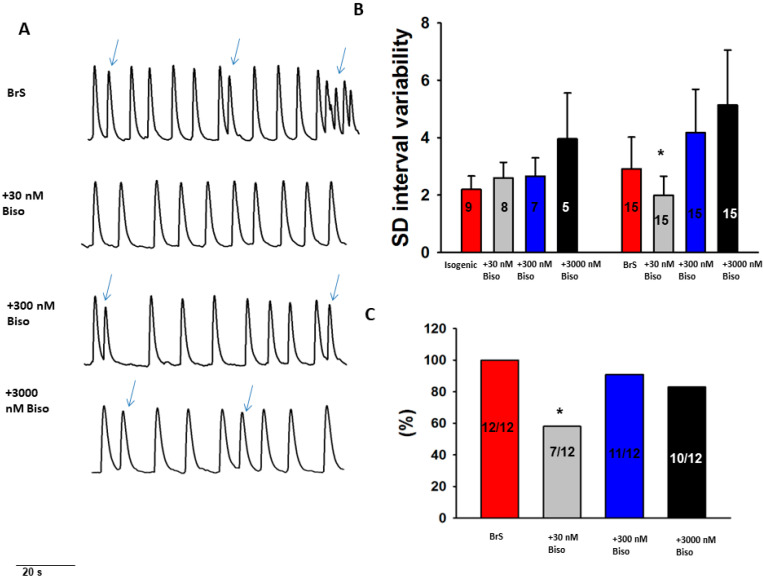
Effects of beta-blocker on the beating variability and arrhythmic events in BrS-hiPSC-CMs. Spontaneous calcium transients were recorded in regularly beating hiPSC-CMs from the BrS patient and the isogenic control(isogenic) in the absence (baseline) and presence of 30 nM, 300 nM and 3000 nM bisoprolol (Biso). The beating variability (standard deviation (SD) of cell beating intervals) and the occurrence of arrhythmic events (irregular or triggered beats or EAD-like events) were compared between with and without drug application. (**A**) Representative trace of BrS (at baseline) and after the administration of different concentrations of bisoprolol. (**B**) Mean values of standard deviation (SD) of cell beating intervals. (**C**) Percentage of cells showing arrhythmic events. * *p* < 0.05 versus isogenic according to Fisher’s exact test (**C**) or versus baseline according to *t*-test (**B**).

**Figure 7:**
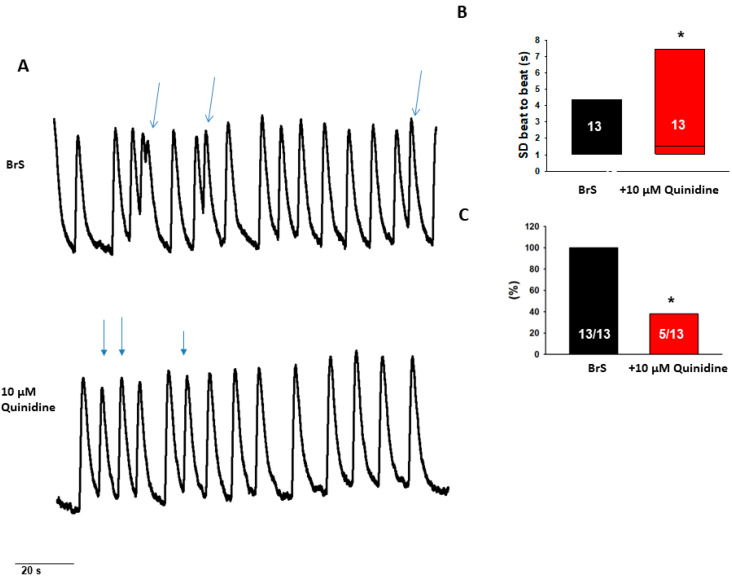
Effects of quinidine on the beating variability and arrhythmic events in BrS-hiPSC-CMs. (**A**) Representative traces of calcium transient before and after administration of 10 µM quinidine in BrS-hiPSC-CMs. Only cells with arrhythmic events were used. (**B**) Median values of standard deviation (SD) of cell beating intervals. (**C**) Percentage of cells showing arrhythmic events. * *p* < 0.05 using Fisher’s exact test (**C**) or versus baseline according to *t*-test (**B**).

To test antiarrhythmic effects of drugs, we examined effects of bisoprolol and quinidine in the BrS-hiPSC-CMs. Bisoprolol (at 30 nM) reduced arrhythmic events and reduced variation in the beat-to-beat interval time (Figure 6). Quinidine reduced only arrhythmic events (Figure 7). These results indicate antiarrhythmic effects of both bisoprolol and quinidine in cardiomyocytes with a CACNB2-variant.

## 3. Discussion

We have, for the first time, generated patient-specific-induced pluripotent stem cell-derived cardiomyocytes (BrS-hiPSC-CMs) from a BrS patient with recurrent SCA carrying a missense variant of uncertain variance in the CACNB2 gene (c.425C > T/p.S142F) and compared their cellular physiological and pharmacological properties with healthy and isogenic control (variant corrected) hiPSC-CMs. Our results regarding BrS-hiPSC-CMs revealed: (i) a reduction of peak L-type calcium current; (ii) reduced protein expression of the CACNB2 gene; (iii) increased arrhythmia-like events in BrS-hiPSC-CMs; and (iv) suppression of arrhythmic events by quinidine and bisoprolol.

It is known that variants in the SCN5A gene may be associated with BrS. This causes a reduction of the peak sodium current. Other genes, e.g., the beta-subunits of the sodium channel have also been related to BrS [5]. Recently published data reported that variants in the alpha-subunit (CACN1C) and beta-subunit (CACNB2) of the L-type calcium channel are linked to BrS and/or short QT syndrome [12,19]. However, a genotype–phenotype correlation of CACNB2 variants with BrS has not been experimentally proven in human cardiomyocytes so far. The present study demonstrated, through functional studies in BrS cells, healthy and isogenic cell lines, that the CACNB2-variant (c.425C > T/p.S142F) is pathogenic for the BrS phenotype (the loss-of-function of the Ca channel and increased arrhythmic activity).

The gene for the human β2 isoform of calcium channel was first identified as a Lambert–Eaton myasthenic syndrome (LEMS) antigen [20] and was mapped to human chromosomes 10p12 [20,21]. The reason for LEMS-patients displaying an immune response against the calcium channel beta subunit is not clear. It is possible that the locus 10p12 of CACNB2 is close to a region undergoing genetic rearrangements during the course of the disease and the expression CACNB2 can be altered, leading to an autoimmune response [20,21]. Whether BrS with CACNB2 mutations is related to LEMS or other autoimmune disorders needs to be explored. LEMS is characterized by the muscle weakness of limbs.

Regarding the functional consequences caused by CACNB2 variants, experimental studies are sparse. A missense variant (T11I) in CACNB2 was reported to lead to the accelerated inactivation of L-type calcium channel currents in TSA201 cells co-transfected with the CACNB2-mutant and wild-type CACNA1C [22]. Another study showed that a polymorphism (D601E) in CACNB2 enhanced the inactivation of L-type calcium currents in TSA201 cells [23]. A study reported that in CHO cells transfected with CACNB2 carrying the variant (S481L) with wild-type CACNA1C, the I_Ca-L_ was reduced [12], indicating that this variant may lead to the loss-of-function of L-type calcium channels. From these studies in non-cardiac cells, however, it cannot be judged whether those variants also suppress I_Ca-L,_ influence action potentials or contribute to the occurrence of arrhythmias in human cardiomyocytes. The heterologous expression cells like CHO and TSA201 cells have a bevy of limitations as compared to human cardiomyocytes and are not suitable for studies mimicking the functions of cardiomyocytes. Therefore, we aimed in the present study to investigate the possible functional roles of the missense variant (c.425C > T/p.S142F) in CACNB2 for the phenotypic characteristics of BrS using hiPSC-CMs. Although this variant has been detected in multiple BrS patients, a recommendation regarding the importance and the clinical significance consistent with the American College of Medical Genetics and Genomics and the Association for Molecular Pathology (ACMG/AMP) and ClinVar classifies the variant as “VUS” (variants with unknown significance) [10,24]. A consequence of change in the normal gene sequence with respect to clinical significance may be “pathogenic”, “likely pathogenic”, “likely benign”, “benign” and “variant of uncertain significance (VUS)”. However, in many cases there is not sufficient information of a VUS to determine its potential significance. Functional studies are important for clarifying potential clinical significance of a VUS. In the current study, we report a case of BrS, in which the disease phenotype was observed as a result of reduced peak L-type calcium current with significant changes in channel expression (protein) level. The reduced calcium current and reduced protein levels resulted from the missense variant in CACNB2, a beta-subunit of the L-type-Ca-channel, as the correction of the variant restored the normal state. To the best of our knowledge, the cellular implications of this variant has not yet been reported, either by in vitro or in vivo studies. Using co-transfected cells two cellular mechanisms with phenotype of BrS in presence of CACNB2 variant have been reported: the accelerated inactivation of L-type-Ca-current (variant: p.T11I) presenting BrS and reduced peak calcium current (variant: p.S481L) presenting an overlap of short QT syndrome and Brugada syndrome [12,22]. In contrast to published data in co-transfected cells, showing that a loss of function variant (p.S481L) in CACNB2 with a reduced peak L-type calcium current [12] presents a novel case of an overlap J-wave disease with shortened QTc, our BrS patient had a normal QTc interval (QTc 400 ms) and the variant S142 in CACNB2 reduced the inactivation and accelerated recovery from the inactivation of L-type calcium channels in the patient’s hiPSC-CMs. An association of J-wave diseases, including BrS with variants of CACNA2D1 and CACNA1C of L-type-calcium channel alpha-subunits, has been reported [19]. Variants of both subunits presented a reduced peak L-type calcium current in heterologous expression cells. Taking all the data together, including the results from the current study, reduced peak calcium current, irrespective of the reasons for the current reduction, can contribute to BrS pathogenesis.

It has been debated that a decrease in I_Na_ [25], I_Ca_ [12] or an increase of outward potassium currents associated with a preferential abbreviation of the action potential as the part of pathophysiological mechanism in BrS leading to heterogeneities of action potential characteristics and a spatial dispersion of repolarization as a dominator for ventricular tachyarrhythmias. However, in our patient the QTc is normal and the APD in hiPSC-CMs from the patient is not shortened, instead slightly prolonged, suggesting that the arrhythmias in the patient did not result from QTc/APD-shortening. The present reduction of I_Ca-L_ could contribute to arrhythmogenesis and might confirm the susceptibility of BrS with variants in CACNB2 to sodium channel blockers as detected in our patient. ECGs of BrS could be modulated by different drugs (e.g., sodium channel blockers) and electrolyte disorders as well as alcohol and cocaine toxicity. Therefore, a consensus suggests the avoidance of sodium channel blockers in BrS [26].

Quinidine, a multiple channel blocker that was tested in some BrS patients, reduced the occurrence of arrhythmic events, suggesting it might be an effective drug for treating BrS with CACNB2 variant. It is of note that our patient has no more ventricular fibrillation after using bisoprolol at low dosage of 2.5 mg per day. Bisoprolol, a beta-blocker, is one of the drugs that should be avoided in BrS patients. However, these data are based either on old literature and/or single case reports. Therefore, we tested the anti-arrhythmic effect of bisoprolol. Indeed, it obviously abolished arrhythmia events at low concentration in hiPSC-CMs from the BrS patient, which is consistent with the clinical data in the patient and again indicates that hiPSC-CMs from the BrS-patient can recapitulate drug effects in the patient. Recently published data showed that patients who were treated with beta-blockers and/or calcium channel antagonists did not suffer from life-threatening arrhythmias at low to normal concentrations [27]. The question could be raised as to whether the drugs, which were supposed to be avoided by BrS patients, might have differential effects in BrS patients, i.e., drug effects could be gene variant associated, with a dosage-dependent effect. This question might be answered in the future by testing drug effects in preclinical cellular models, such as hiPSC-CMs harboring different gene variants. In summary, the hiPSC-CMs from the patient with the CACNB2 variant displayed the phenotypic features of BrS and can be used as a platform for further mechanistic or therapeutic studies for BrS. In the future, patient-specific hiPSC-CMs may provide a powerful platform for studying the pathogenic roles of different gene mutations identified in BrS patients. Furthermore, hiPSC-CMs may be especially helpful for personalized medicine, such as personalized drug testing or other personalized treatment strategy.

## 4. Materials and Methods

### 4.1. Ethics Statement

The skin biopsies from three healthy donors and one BrS patient were obtained with written informed consent. The study was approved by the Ethics Committee of the Medical Faculty Mannheim, University of Heidelberg (approval number: 2018-565N-MA) and by the Ethics Committee of University Medical Center Göttingen (approval number: 10/9/15). The study was carried out in accordance with the approved guidelines and conducted in accordance with the Helsinki Declaration of 1975, as revised in 1983.

### 4.2. DNA-Sequencing Analysis

DNA was isolated from blood lymphocytes. Ninety-eight of one hundred and seven coding exons of the genes CACNA1C, CACNB2, GPD1L, KCNE3, SCN1B, SCN3B and SCN5A including exon/intron boundaries were amplified by PCR and subjected to bidirectional Sanger sequencing. Sequences were mapped against hg19 references (NM_015141.3, NM_000719.5, NM_201590.2, NM_001037.4, NM_005472.4, NM_018400.3 and NM_198056.2) via JSI Sequence Pilot for further analysis.

### 4.3. Generation of Human iPS Cells

Human iPS cells (hiPSCs) were generated from primary human fibroblasts derived from skin biopsies. The three healthy cell lines (D1, UMGi014-B and UMGi124-A) have been described previously [28,29]. The BrS cell line was generated in feeder free culture conditions using the integration-free CytoTune-iPS 2.0 Sendai Reprogramming Kit (Thermo Fisher Scientific, Waltham, MA, USA, #A16517) with the reprogramming factors OCT4, KLF4, SOX2 and c-MYC according to manufacturer’s instructions with modifications, as described previously [30]. The generated hiPSCs (isBrSb2.1/UMGi119-A.1 and isBrSb2.2/UMGi119-A.2) were characterized for their pluripotency.

### 4.4. Gene-Editing

Isogenic gene-corrected control iPSC lines (isBrSb2-corr.6/UMGi119-A-1.6/BIHi259-A-1 and isBrSb2-corr.23/UMGi119-A-1.23/BIHi259-A2) were generated using a protocol previously described (Christopher D Richardson et al., 2016). In brief, small guide RNA (sgRNA) targeting the CACNB2 gene close to the variant site to be corrected were designed using the CRIPSOR online tool (Concordet and Haeussler 2018 [31]). The donor ssODN template was designed to correct the disease-causing variant (c428T > C) and was synthesized as an Ultramar DNA oligo by IDT. (5′-GCTGCAGCATGAACAGAGAGCCAAGCAAGGGAAATTCTACTCCAGGTATGAGACAGATGTCAAGTGTTTGCATAAAACTTAGATTATACAACTAGCTGTGTACTGTTGTCTGCTGTATTCTGTATCC-3′). By correcting the specific variant, we generated an Alu1 restriction enzyme recognition site which was used later on for the screening and identification of corrected clones. The Ribonucleoprotein (RNP) complexes were prepared by the mixing and incubation of 1.5 µg Cas-9 protein and 360 ng gRNA for 10 min at room temperature. For delivery of RNPs and ssODN template, 10 µL cell suspension containing Cas9 RNPs and ssODN and 1 × 10^5^ cells were electroporated using the Neon transfection System 10 µL Kit (Thermo Fisher Scientific, Cat. No. MPK1025) and the following protocol: 1200 V, 30 ms pulse, 1 pulse. The electroporated cells were plated in one well of 6 well plate with StemFlex media (Thermo Fisher Scientific, Cat. No. A3349401) supplemented CloneR™ (Stemcell Technologies, Vancouver, BC, Canada, Cat. No. 05888). Three days after the transfection we analyzed the bulk population using the Amplicon Sequencing Service from Genewiz (Amplicon-EZ) to estimate the editing efficiency (https://www.genewiz.com). Thereafter, the automated single cell cloning of the genome edited cell pool was performed as described in protocol (Fernandez Vallone and Narasimha Telugu et al. 2020). The clones were screened by performing Alu1 restriction digestion analysis and the positive clones were validated by SANGER sequencing. The positive confirmed clones were banked and characterized for pluripotency and karyotype stability.

### 4.5. Differentiation of Human iPS Cells into Cardiomyocytes

Feeder-free hiPSCs were thawed and differentiated into hiPSC-CMs, as described with some modifications in [30]. At 50–60 days of culture with basic culture medium, cardiomyocytes were dissociated from well plates and plated on Matrigel-coated 3.5 cm petri dishes for the experiments.

### 4.6. Polymerase-Chain-Reaction Assays

To quantify the steady-state mRNA expression of the hiPSC-CMs, RNA was reverse transcribed and qPCR was performed, as described in [32]. Gene symbols, RefSeq No. and Cat. No. of the primers used for qPCR analyses in hiPSC-CMs characterization were listed in Supplementary Table S1.

### 4.7. Immunofluorescence Staining

All antibodies used for the characterization of hiPSC-CMs are listed in Supplementary Table S2.

### 4.8. Western Blot

All antibodies used for the Western blot analysis of hiPSC-CMs are listed in Supplementary Table S2.

### 4.9. Drugs

Using a perfusion pipette different drugs (ajmaline, bisoprolol and carbachol) were applied to a hiPSC-CM. The tested concentrations were selected according to previous or our preliminary studies in hiPSC-CMs. Ajmaline (MP Biomedicals, Irvine, CA, USA) was dissolved in DMSO at a stock concentration of 30 mM. Bisoprolol was dissolved in water at a stock concentration of 3 mM. Quinidine (Sigma, Setagaya City, Tokyo) was dissolved in water at 10 mM of stock solution.

### 4.10. Patch Clamp and Calcium Transient Measurements

The methods of patch clamp and calcium transient measurements were carried out as described in previously publications [33,34].

### 4.11. Statistical Analysis

Data are shown as mean ± SEM in case of normal distribution or mean ± SD in case of non-normal distribution and were analyzed using InStat© (GraphPad, San Diego, SC, USA) and SigmaPlot 11.0 (Systat GmbH, Erkrath, Germany). The normal distribution of data was assessed using the Shapiro–Wilk test. For normally distributed data of two or more than two groups, *t*-test or multiple comparisons with one-way ANOVA and the Holm–Sidak post-test were performed. For non-normally distributed data, the Mann–Whitney rank sum rest (two groups) or the Kruskal–Wallis one way analysis of variance on ranks and Dunn´s method for post-test (>two groups) were used. Paired *t*-test was used for comparisons of data before and after the application of a drug. To compare categorical variables, the Fisher test was used. *p* < 0.05 (two-tailed) was considered significant.

### 4.12. Western Blot

Cells were collected and sonicated in RIPA buffer (R0278, Merck KGaA, Darmstadt, Germany) and the protein concentration was detected by BCA Protein Assay Kit (23227, Thermo Fisher Scientific, Waltham, MA, USA). The protein samples were loaded 20 μg per sample for SDS-PAGE and then transferred to the PVDF membrane (IPVH00010, Merck KGaA, Darmstadt, Germany). After blocking with 5% nonfat milk for 1 h at room temperature, the membranes were incubated with the primary antibodies at 4 °C overnight and then with secondary antibodies at room temperature for 1 h (shown in Supplement Table S4). The target protein bands were quantified by the Fusion Solo system (Vilber, Collégien, France) and measured with software (Image J software, Research Services Branch, National Institute of Mental Health, Bethesda, MD, USA) for statistical analyses.

### 4.13. Immunostaining

Cells were washed by phosphate-buffered saline (PBS) and fixed by 4% paraformaldehyde for 10 min, incubated in membrane penetration buffer (0.1% Triton X-100 in PBS) for 10 min and blocked in blocking solution (5% fetal bovine serum) for 30 min. Then the cells were incubated with primary antibodies (shown in Supplementary Tables S3 and S4) overnight at 4 °C and with the second antibodies (shown in Supplementary Tables S3 and S4) in room temperature in the dark for 1 h. Pictures were captured by the fluorescence microscope (Leica DMRE, Leica Mikrosysteme Vertrieb GmbH, Wetzlar, Germany) and analyzed by Image J software (Research Services Branch, National Institute of Mental Health, Bethesda, MD, USA).

## 5. Conclusions

Our data demonstrate that the missense variant (c.425C > T/p.S142F) in CACNB2 is pathogenic for BrS. The phenotypic features of BrS patients in presence of CACNB2 variant were successfully recapitulated in the hiPSC-CMs, including reduced peak I_Ca-L_, reduced protein level of CACNB2 and increased arrhythmia events at baseline. Bisoprolol and quinidine might be effective drugs in BrS patients with variants in the CACNB2 gene.

### 5.1. Study Limitations

In addition to similarities, hiPSC-CMs present also differences in their properties as compared to adult human cardiomyocytes. Immature features including spontaneous beats, lack of some structural and functional proteins and depolarized membrane potential are well-known differences comparing with adult human cardiomyocytes. These differences may be critical for studies, especially when studies are related to signaling that are different in hiPSC-CMs and human adult cardiomyocytes. For example, the expression of the inward rectifier potassium (IK1) channel in hiPSC-CMs is low, which is the main reason for the depolarization. Studies on IK1-related signaling may display different results in hiPSC-CMs and human adult cardiomyocytes. Of note, many factors, such as nerve and hormone interactions with cardiomyocytes are not taken into consideration. Due to the difficulty of finding and recruiting BrS patients with identical gene variants, only one patient was included in this study. Individual variability cannot be excluded, but the patient-specific study has also clinical significance, especially for precision medicine.

### 5.2. Translational Perspective

Brugada syndrome (BrS) is a rare inherited channelopathy. It is difficult to obtain cardiomyocytes from BrS patients for mechanistic or therapeutic studies. This study demonstrated that induced pluripotent stem cell-derived cardiomyocytes (hiPS-CMs) from BrS form 4, carrying a CACNB2-variant with unclear significance recapitulated the cellular phenotype of BrS patients. Therefore, the patient-specific hiPSC-CMs may provide a good research platform to gain new insights into the cellular mechanisms of BrS 4 and establish a personalized medicine for patients.

## Data Availability

Required data will be available by asking the corresponding author.

## Supplementary Materials

The following supporting information can be downloaded at: https://www.mdpi.com/article/10.3390/ijms23158313/s1.
