# Supplementary material for: A Preclinical Study on Brugada Syndrome with a CACNB2 Variant Using Human Cardiomyocytes from Induced Pluripotent Stem Cells
Source: Int J Mol Sci. 2022 Jul 27;23(15):8313. doi: 10.3390/ijms23158313 (PMC9368582; doi:10.3390/ijms23158313)
Supplement: Supplementary file 1 [file ijms-23-08313-s001.zip › ijms-1790496-supplementary.pdf]

Supplementary Data

A Preclinical Study on Brugada Syndrome with a CACNB2 Variant Using Human Cardiomyocytes from Induced Pluripotent Stem Cells

Rujia Zhong [1,†], Theresa Schimanski [1,2,†], Feng Zhang [1], Huan Lan [1,2,3], Alyssa Hohn [1,2], Qiang Xu [1], Mengying Huang [1], Zhenxing Liao [1], Lin Qiao [1], Zhen Yang [1], Yingrui Li [1], Zhihan Zhao [1,2], Xin Li [1], Lena Rose [1], Sebastian Albers [1,2], Lasse Maywald [1,2], Jonas Müller [1,2], Hendrik Dinkel [1,2], Ardan Saguner [4], Johannes W. G. Janssen [5], Narasimha Swamy [2,6], Yannick Xi [1], Siegfried Lang [1], Mandy Kleinsorge [2,7], Firat Duru [4], Xiaobo Zhou [1,2,3,*], Sebastian Diecke [2,6,‡], Lukas Cyganek [2,7,‡], Ibrahim Akin [1,2,‡] and Ibrahim El-Battrawy [8,‡]

1 First Department of Medicine, Faculty of Medicine, University Medical Centre Mannheim (UMM), University of Heidelberg, 68167 Mannheim, Germany; rujia.zhong@medma.uni-heidelberg.de (R.Z.); schimanski.t@gmail.com (T.S.); feng.zhang@medma.uni-heidelberg.de (F.Z.); huan.lan@medma.uni-heidelberg.de or lh6402196@126.com (H.L.); alyssa.hohn@web.de (A.H.); qiang.xu@medma.uni-heidelberg.de (Q.X.); mengying.huang@medma.uni-heidelberg.de (M.H.); zhenxing.liao@medma.uni-heidelberg.de (Z.L.); lin.qiao@medma.uni-heidelberg.de (L.Q.); zhen.yang@medma.uni-heidelberg.de (Z.Y.); yingrui.li@medma.uni-heidelberg.de (Y.L.); zhihan.zhao@medma.uni-heidelberg.de (Z.Z.); xin.li@medma.uni-heidelberg.de (X.L.); roselena1996@gmail.com (L.R.); sebastian9876@googlemail.com (S.A.); lasse-maywald@web.de (L.M.); jonasnelsonmueller@googlemail.com (J.M.); hendrik.dinkel@yahoo.de (H.D.); yannick.xi@medma.uni-heidelberg.de (Y.X.); siegfried.lang@umm.de (S.L.); ibrahim.akin@umm.de (I.A.)

2 DZHK (German Center for Cardiovascular Research), Partner Site, 68167 Mannheim, Germany; narasimha.swamy@mdc-berlin.de (N.S.); mandy.kleinsorge@gwdg.de (M.K.); sebastian.dieck@mdc-berlin.de (S.D.); lukas.cyganek@gwdg.de (L.C.)

3 Key Laboratory of Medical Electrophysiology of Ministry of Education and Medical Electrophysiological Key Laboratory of Sichuan Province, Institute of Cardiovascular Research, Southwest Medical University, Luzhou 646000, China

4 Department of Cardiology, University Heart Center Zurich, Rämistrasse 100, 8091 Zürich, Switzerland; ardan.saguner@usz.ch (A.S.); first.duru@usz.ch (F.D.)

5 Department of Human Genetics, Institute of Human Genetics, University of Heidelberg, 69120 Heidelberg, Germany; johannes.jannsen@uni-heidelberg.de

6 Max Delbrück Center for Molecular Medicine, 13125 Berlin, Germany

7 Stem Cell Unit, Clinic for Cardiology and Pneumology, University Medical Center Göttingen, 37075 Göttingen, Germany

8 Bergmannsheil University Hospitals, Ruhr University of Bochum, 44789 Bochum, Germany; ibrahim.elbattrawy2006@gmail.com

* Correspondence: xiaobo.zhou@medma.uni-heidelberg.de; Tel.: +49-621-383-1448; Fax: +49-621-383-1474

† These authors contributed equally to this work.

‡ These authors share the senior authorship.

Material and Methods

Ethics statement

The skin biopsies from three healthy donors and one BrS patient were obtained with written informed consent. The study was approved by the Ethics Committee of the Medical Faculty Mannheim, University of Heidelberg (approval number: 2018-565N-MA) and by the Ethics Committee of University Medical Center Göttingen (approval number: 10/9/15). The study was carried out in accordance with the approved guidelines and conducted in accordance with the Helsinki Declaration of 1975, as revised in 1983.

DNA-sequencing analysis

DNA was isolated from blood lymphocytes. Ninety-eight of 107 coding exons of the genes CACNA1C, CACNB2, GPD1L, KCNE3, SCN1B, SCN3B and SCN5A including exon/intron boundaries were amplified by PCR and subjected to bidirectional Sanger sequencing. Sequences were mapped against hg19 references (NM_015141.3, NM_000719.5, NM_201590.2, NM_001037.4, NM_005472.4, NM_018400.3 and NM_198056.2) via JSI Sequence Pilot for further analysis.

Generation of human iPS cells

Human iPS cells (hiPSCs) were generated from primary human fibroblasts derived from skin biopsies. The three healthy cell lines (D1, UMGi014-B and UMGi124-A) have been described previously [1, 2]. The BrS cell line was generated in feeder free culture conditions using the integration-free CytoTune-iPS 2.0 Sendai Reprogramming Kit (Thermo Fisher Scientific, #A16517) with the reprogramming factors OCT4, KLF4, SOX2, c-MYC according to manufacturer's instructions with modifications as described previously [3]. The generated hiPSCs (isBrSb2.1/UMGi119-A.1 and isBrSb2.2/UMGi119-A.2) were characterized for their pluripotency.

Gene-editing

Isogenic gene-corrected control iPSC lines (isBrSb2-corr.6/UMGi119-A-1.6/ BIHi259-A-1 and isBrSb2-corr.23/UMGi119-A-1.23/ BIHi259-A2) were generated using a protocol previously described (Christopher D Richardson et al 2016). In brief, small guide RNA (sgRNA) targeting the CACNB2 gene close to the variant site to be corrected were designed using the http://crispor.tefor.net/ webpage and the sgRNA (5`-AGCAAGGGAAATTCTACTTC-3`) were synthesized by Integrated DNA technologies (https://eu.idtdna.com). The donor ssODN template was designed to correct the disease causing variant (c428T>C) and got synthesized as an Ultramar DNA

oligo by IDT. (5′-GCTGCAGCATGAACAGAGAGCCAAGCAAGGGAAATTCTACTCCAGGTATGAGACAGATGTCAAGTGTTTGCATAAAACTTAGATTATACAACTAGCTGTGTACTGTTGTCTGCTGTATTCTGTATCC-3′). By correcting the specific variant we generated an Alu1 restriction enzyme recognition site which were used later on for the screening and identification of corrected clones. The Ribonucleoprotein (RNP) complexes were prepared by mixing and incubation of 1.5µg Cas-9 protein and 360 ng gRNA for 10 minutes at room temperature. For delivery of RNPs and ssODN template, 10µl cell suspension containing Cas9 RNPs and ssODN and $1x10^5$ cells were electroporated using the Neon transfection System 10µl Kit (ThermoFisher Scientific, Cat. No. MPK1025), and the following protocol 1200V, 30ms pulse, 1 pulse. The electroporated cells were plated in one well of 6 well plate with StemFlex media (ThermoFisher Scientific, Cat.No. A3349401) supplemented CloneR™ (Stemcell technologies, Cat. No. 05888). Three days after the transfection we analyzed the bulk population using the Amplicon Sequencing Service from Genewiz (Amplicon-EZ) to estimate the editing efficiency (https://www.genewiz.com). Thereafter, the automated single cell cloning of the genome edited cell pool was performed as described in protocol (Fernandez Vallone and Narasimha Telugu et al 2020). The clones were screened by performing Alu1 restriction digestion analysis and the positive clones were validated by SANGER sequencing. The positive confirmed clones were banked and characterized for pluripotency and karyotype stability.

Differentiation of human iPS cells into cardiomyocytes

Feeder free hiPSCs were thawed and differentiated into hiPSC-CMs as described with some modifications [4]. At 50-60 days of culture with basic culture medium, cardiomyocytes were dissociated from well plates and plated on Matrigel-coated 3.5 cm petri dishes for the experiments.

Polymerase-Chain-Reaction Assays

To quantify the steady-state mRNA expression of the hiPSC-CMs, RNA was reverse transcribed and qPCR was performed as described [5]. Gene symbols, RefSeq No. and Cat. No. of the primers used for qPCR analyses in hiPSC-CMs characterization were listed in supplementary Table S1.

Immunofluorescence staining

All antibodies used for characterization of hiPSC-CMs are listed in supplementary Table S2.

Western blot

All antibodies used for western blot of hiPSC-CMs are listed in supplementary Table S2.

Drugs

Using a perfusion pipette different drugs (ajmaline, bisoprolol and carbachol) were applied to a hiPSC-CM. The tested concentrations were selected according to previous or our preliminary studies in hiPSC-CMs. Ajmaline (MP Biomedicals) was dissolved in DMSO at a stock concentration of 30 mM. Bisoprolol was dissolved in water at a stock concentration of 3 mM. Quinidine (Sigma) was dissolved in water at10 mM stock solution.

Patch clamp and calcium transient measurements

The methods of patch clamp and calcium transient measurements have been carried out as described in previously publications [1, 6].

Statistical analysis

Data are shown as mean ± SEM and were analyzed using InStat© (GraphPad, San Diego, USA) and SigmaPlot 11.0 (Systat GmbH, Germany). For data of more than two groups multiple comparisons with one-way ANOVA and Holm-Sidak post-test were performed. Paired t-test was used for comparisons of data before and after application of a drug. To compare categorical variables, the Fisher-test was used. $p<0.05$ (two-tailed) was considered significant.

Western blot

Cells were collected and sonicated in RIPA buffer (R0278, Merck KGaA, Darmstadt, Germany) and the protein concentration was detected by BCA Protein Assay Kit (23227, Thermo Fisher Scientific, Waltham, MA, USA). The protein samples were loaded 20μg per sample for SDS-PAGE, and then transferred to the PVDF membrane (IPVH00010, Merck KGaA, Darmstadt, Germany). After blocking with 5% nonfat milk for 1h at room temperature, the membranes were incubated with the primary antibodies (shown in supplementary Table S4) at 4°C overnight and then secondary antibodies in room temperature for 1h (shown in supplement Table S4). The target protein bands were quantified by Fusion Solo system (Vilber, France) and measured with software (Image J software, Research Services Branch, National Institute of Mental Health, Bethesda, Maryland, USA) for statistical analyses.

Immunostaining

Cells were washed by phosphate-buffered saline (PBS) and fixed by 4% paraformaldehyde for 10 minutes, incubated in membrane penetration buffer (0.1% Triton X-100 in PBS) for 10 minutes and blocked in blocking solution (5% fetal bovine serum) for 30 minutes. Then the cells were incubated with primary antibodies (shown in supplementary Table S4) overnight at 4 °C and the second antibodies (shown in supplementary Table S4) in room temperature in the dark for 1h. Pictures were captured by the fluorescence microscope (Leica DMRE, Leica Mikrosysteme Vertrieb GmbH, Wetzlar, Germany) and analyzed by Image J software (Research Services Branch, National Institute of Mental Health, Bethesda, Maryland, USA).

References

1. El-Battrawy I, Lan H, Cyganek L, Zhao Z, Li X, Buljubasic F, Lang S, Yucel G, Sattler K, Zimmermann WH, Utikal J, Wieland T, Ravens U, Borggrefe M, Zhou XB and Akin I. Modeling Short QT Syndrome Using Human-Induced Pluripotent Stem Cell-Derived Cardiomyocytes. *Journal of the American Heart Association*. 2018;7.
2. El-Battrawy I, Muller J, Zhao Z, Cyganek L, Zhong R, Zhang F, Kleinsorge M, Lan H, Li X, Xu Q, Huang M, Liao Z, Moscu-Gregor A, Albers S, Dinkel H, Lang S, Diecke S, Zimmermann WH, Utikal J, Wieland T, Borggrefe M, Zhou X and Akin I. Studying Brugada Syndrome With an SCN1B Variants in Human-Induced Pluripotent Stem Cell-Derived Cardiomyocytes. *Front Cell Dev Biol*. 2019;7:261.
3. Hanses U, Kleinsorge M, Roos L, Yigit G, Li Y, Barbarics B, El-Battrawy I, Lan H, Tiburcy M, Hindmarsh R, Lenz C, Salinas G, Diecke S, Muller C, Adham I, Altmuller J, Nurnberg P, Paul T, Zimmermann WH, Hasenfuss G, Wollnik B and Cyganek L. Intronic CRISPR Repair in a Preclinical Model of Noonan Syndrome-Associated Cardiomyopathy. *Circulation*. 2020;142:1059-1076.
4. Cyganek L, Tiburcy M, Sekeres K, Gerstenberg K, Bohnenberger H, Lenz C, Henze S, Stauske M, Salinas G, Zimmermann WH, Hasenfuss G and Guan K. Deep phenotyping of human induced pluripotent stem cell-derived atrial and ventricular cardiomyocytes. *JCI Insight*. 2018;3.
5. El-Battrawy I, Lang S, Zhao Z, Akin I, Yucel G, Meister S, Patocskai B, Behnes M, Rudic B, Tulumen E, Liebe V, Tiburcy M, Dworacek J, Zimmermann WH, Utikal J, Wieland T, Borggrefe M and Zhou XB. Hyperthermia Influences the Effects of Sodium Channel Blocking Drugs in Human-Induced Pluripotent Stem Cell-Derived Cardiomyocytes. *PloS one*. 2016;11:e0166143.
6. El-Battrawy I, Zhao Z, Lan H, Li X, Yucel G, Lang S, Sattler K, Schunemann JD, Zimmermann WH, Cyganek L, Utikal J, Wieland T, Bieback K, Bauer R, Ratte A, Pribe-Wolferts R, Rapti K, Nowak D, Wittig J, Thomas D, Most P, Katus HA, Ravens U, Schmidt C, Borggrefe M, Zhou XB, Muller OJ and Akin I. Ion Channel Dysfunctions in Dilated Cardiomyopathy in Limb-Girdle Muscular Dystrophy. *Circ Genom Precis Med*. 2018;11:e001893.

Supplementary Table S1. Gene symbols, RefSeq No. and Cat. No. of the primers used for ion channel qPCR.

Gene symbol	RefSeq No.	Cat. No. Primers
CACNA1C (L-type Ca2+ channel)	NM_000719	PPH01378G
CACNb2	NM_000724	QT00011256
KCND3 (Ito, Kv4.3)	NM_004980	PPH06923A
KCNH2 (IKr, Kv11.1)	NM_000238	PPH01660A
KCNJ2	NM_000891	PPH01618E
KCNQ1 (I_{Ks}, Kv7.1)	NM_000218	PPH01419A
KCNIP2	NM_014591	QT00016254
POU5F1	NM_002701.5	PPH02394E
SCN1B	NM_001037	QT00066080
SCN3B	NM_018400	PPH07274A
SCN5A (Na+ channel, Nav1.5)	NM_000335	PPH01671F
SCN10A (Na+ channel, Nav1.8)	NM_006514	PPH15064A
TNNT2	NM_000364	PPH02619A

RefSeq No. : GenBank NCBI Reference Sequences

Cat. No. Primers: Qiagen RT² qPCR Primer Assays

Supplementary Table S2. Primer sequences, annealing temperatures and cycles used for RT-PCR analyses in iPSC pluripotency characterization.

Primer	Primer for	Primer rev	Length bps	Temp /Cycles
OCT4	GACAACAATGAAAATCTTCAGGAGA	TTCTGGCGCCGGTTACAGAACCA	218	58°C / 36 cycles
SOX2	ATGCACCGCTACGACGTGA	CTTTTGCACCCCTCCCATTT	437	58°C / 30 cycles
NANOG	AGTCCCAAAGGCAAACAACCCACTTC	ATCTGCTGGAGGCTGAGGTATTTCTGTCTC	164	64°C / 36 cycles
LIN28	AGTAAGCTGCACATGGAAGG	ATTGTGGCTCAATTCTGTGC	410	58°C / 36 cycles
FOXD3	GTGAAGCCGCCTTACTCGTAC	CCGAAGCTCTGCATCATGAG	353	58°C / 38 cycles
GDF3	TTCGCTTTCTCCCAGACCAAGGTTTC	TACATCCAGCAGGTTGAAGTGAACAGCACC	311	58°C / 32 cycles
GAPDH	AGAGGCAGGGATGATGTTCT	TCTGCTGATGCCCCCATGTT	258	58°C / 30 cycles

Supplementary Table S3. Antibodies and dilutions used for immunocytochemistry of iPSC pluripotency characterization.

**Primary antibody**	**Type**	**Dilution**	**Supplier**
hOCT3/4	polyclonal goat IgG	1:40	R&D Systems, #AF1759
hSOX2	monoclonal mouse IgG1	1:200	Thermo Fisher Scientific, #MA1-014
hNANOG	polyclonal rabbit IgG	1:100	Thermo Fisher Scientific, #PA1-097
hLIN28	polyclonal goat IgG	1:300	R&D Systems, #AF3757
hSSEA4	monoclonal mouse IgG3	1:100	Thermo Fisher Scientific, #MA1-021
hTRA-1-60	monoclonal mouse IgM	1:200	Abcam, #ab16288
hAFP	polyclonal rabbit IgG	1:100	DAKO, #A0008
α-SMA	monoclonal mouse IgG2a	1:3000	Sigma-Aldrich, #A2547
β-III-Tubulin	monoclonal mouse IgG2A	1:2000	Covance, #MMS-435P
**Secondary antibody**	**Type**	**Dilution**	**Supplier**
Alexa Fluor 488	polyclonal donkey α-mouse IgG	1:1000	Thermo Fisher Scientific, #A21202
Alexa Fluor 555	polyclonal donkey α-goat IgG	1:1000	Thermo Fisher Scientific, #A21432
Alexa Fluor 555	polyclonal donkey α-mouse IgG	1:1000	Thermo Fisher Scientific, #A31570
Alexa Fluor 555	polyclonal donkey α-rabbit IgG	1:1000	Thermo Fisher Scientific, #A31572
FITC	polyclonal goat α-mouse IgM	1:200	Jackson Immuno, #115-097-020
**Flow Cytometry**		**Dilution**	**Supplier**
Alexa Fluor 488 mouse anti-human TRA-1-60		1:50	BD Biosciences, #560173
Alexa Fluor 647 mouse anti-OCT3/4		1:50	BD Biosciences, #560329

Supplementary Table S4. Antibodies and dilutions used for immunocytochemistry and western blot analysis in cardiomyocytes derived from iPSC cell culture.

**Primary antibody**	**Type**	**Dilution**	**Supplier**
α-Actinin	monoclonal mouse IgG1	1:400 (IF)	Sigma-Aldrich, A7811
TnT	monoclonal mouse IgG1	1:200 (IF)	Abcam, ab8295
SCN5A	monoclonal mouse IgM	1:200 (IF), 1:500 (WB)	Abcam, ab62388
CACNB2	polyclonal rabbit IgG	1:200 (IF)	ATLAS, HPA035326
CACNB2	monoclonal mouse IgG1	1:500 (WB)	Abcam, ab54920
GAPDH	monoclonal mouse IgG	1:100000 (WB)	HyTest, 5G4
**Secondary antibody**	**Type**	**Dilution**	**Supplier**
Alexa Fluor 568	polyclonal goat anti-rabbit IgG	1:1000 (IF)	Thermo Fisher Scientific, A-11036
Alexa Fluor 488	polyclonal goat anti-mouse IgG	1:1000 (IF)	Thermo Fisher Scientific, A32723
Anti-rabbit IgG	polyclonal goat anti rabbit IgG-peroxidase	1:2000 (WB)	Sigma-Aldrich, A0545
Anti-mouse IgG	polyclonal goat anti mouse IgG-peroxidase	1:2000 (WB)	Sigma-Aldrich, A3682

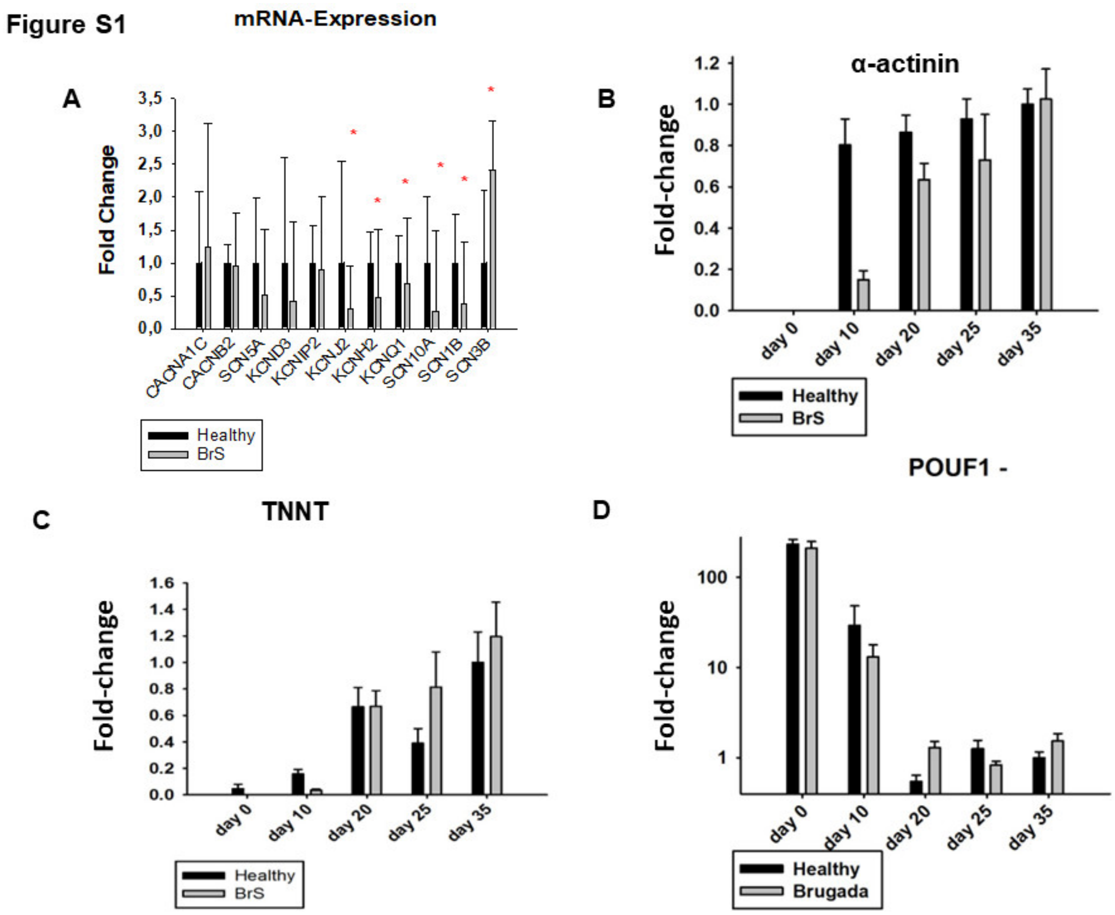

Figure S1. Expression of cardiac markers and ion channels in hiPSC-CMs. qPCR-analysis was performed to analyze the mRNA levels of cardiac markers TNNT2 and alpha-actinin and a pluripotency marker POUF1, in addition to different ion channels. The mRNA levels were compared between donor (Healthy) and the patient (BrS) cell line. (A) mRNA levels of ion channels including calcium channels (CACNA1C, CACNB2), sodium channels (SCN5A, SCN10A, SCN1B and SCN3B) and potassium channels (KCND3, KCHIP, KCNJ2, KCNH2 and KCNQ1) in hiPSC-CMs at day 35. (B) mRNA levels of alpha-actinin in cells at the start (day 0) and different time points of differentiation of hiPSCs into cardiomyocytes. (C) mRNA levels of TNNT2 in cells at the start (day 0) and different time points of differentiation of hiPSCs into cardiomyocytes. (D) mRNA levels of POUF1 in cells at the start (day 0) and different time points of differentiation of hiPSCs into cardiomyocytes. * $p < 0.05$ versus healthy analyzed by t-test for two groups.

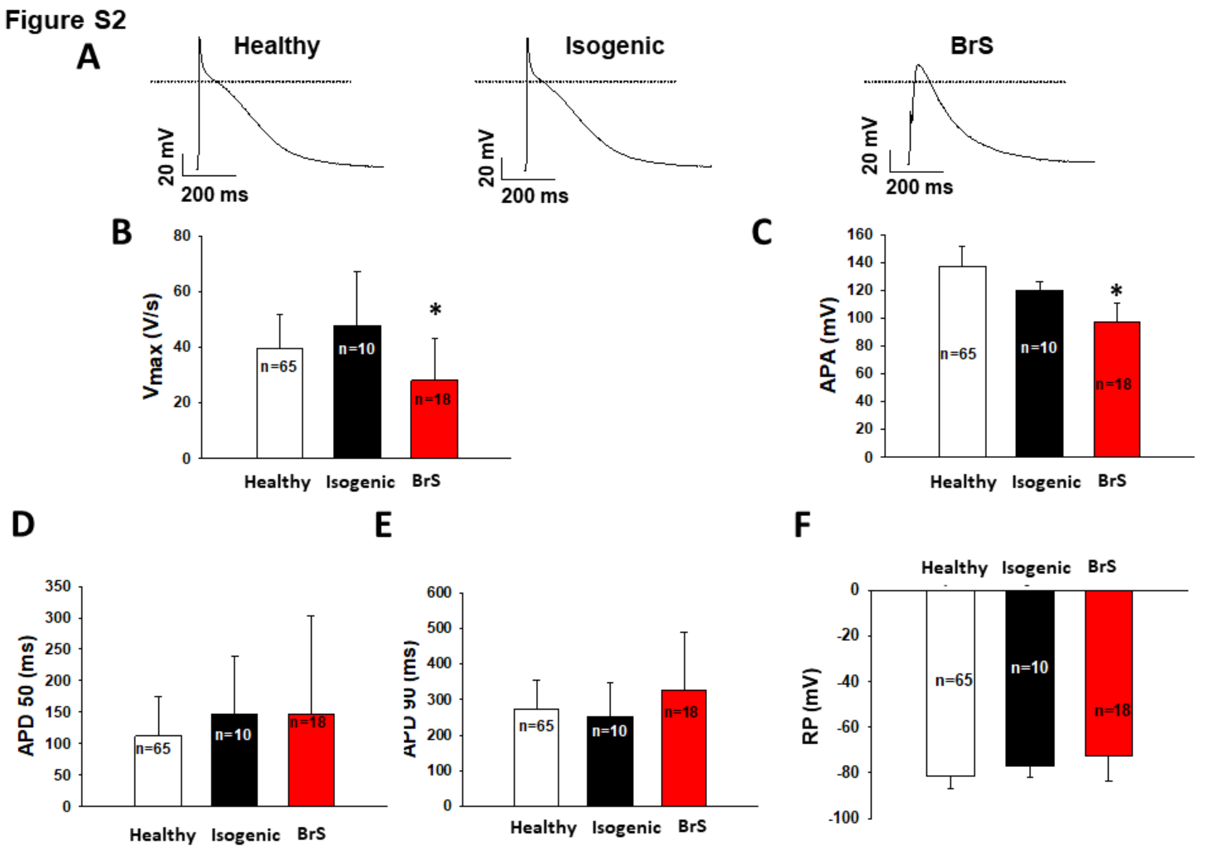

Figure S2. Abnormal action potentials in hiPSC-CMs from the BrS patient. Action potentials paced at 1 Hz were recorded by patch clamp techniques in hiPSC-CMs from healthy donors (Healthy), the BrS patient (BrS) and CRISPR/Cas9-corrected BrS cells (Isogenic). **(A)** Representative AP-traces recorded in a cell from each cell line at 1 Hz. **(B)** Averaged values of maximal depolarization velocity (Vmax) in each cell line. **(C)** Averaged values of action potential amplitude (APA) in each cell line. **(D-E)** Averaged values of action potential duration at 50% (APD50) and 90% (APD90) in each cell line at 1 Hz.**(F)** Averaged values of resting potential (RP) in each cell line at 1 Hz. Numbers given in B represent the number of cells for B-F. * $p < 0.05$ versus Healthy according to one-way ANOVA with Holm-Sidak post-test.
